# Supplementary material for: Characterization of the cholangiocarcinoma drug pemigatinib against FGFR gatekeeper mutants
Source: Commun Chem. 2022 Aug 22;5:100. doi: 10.1038/s42004-022-00718-z (PMC9814635; doi:10.1038/s42004-022-00718-z)
Supplement: Supplementary file 2 — Supplementary Material [file 42004_2022_718_MOESM2_ESM.pdf]

Supplementary Tables and Figures

Supplementary Table 1. Data collection and refinement statistics

	FGFR1/Pemigatinib
**Data collection**	
Space group	C 1 21 1
Cell dimensions	
a, b, c (Å)	210.83, 49.59, 66.80
α, β, γ (°)	90.00, 107.50, 90.00
Resolution (Å)	39.87 – 2.495 (2.584 – 2.495)
R_{sym} or R_{merge}	0.06982/ 0.07907
$I / \sigma I$	15.13 (3.05)
Completeness (%)	93.45 (61.78)
Redundancy	4.5 (3.0)
**Refinement**	
Resolution (Å)	39.87 – 2.495
No. reflections	97398 (4208)
R_{work} / R_{free}	0.19/0.23
No. atoms	
Protein	593
Ligand/ion	100
Water	90
B-factors	
Protein	44.50
Ligand/ion	48.10
Water	40.00
R.m.s. deviations	
Bond lengths (Å)	0.004
Bond angles (°)	0.78

Supplementary Table 2. Predicted binding energies of FGFRs and gatekeeper mutants with pemigatinib.

	Predicted Binding Energy (Kcal·mol^{-1})
FGFR1	-11.80
FGFR2	-11.17
FGFR3	-10.92
FGFR4	-10.86
FGFR1 V561M	-6.53
FGFR2 V564I	-10.71
FGFR2 V564F	-8.65
FGFR3 V555M	-9.29

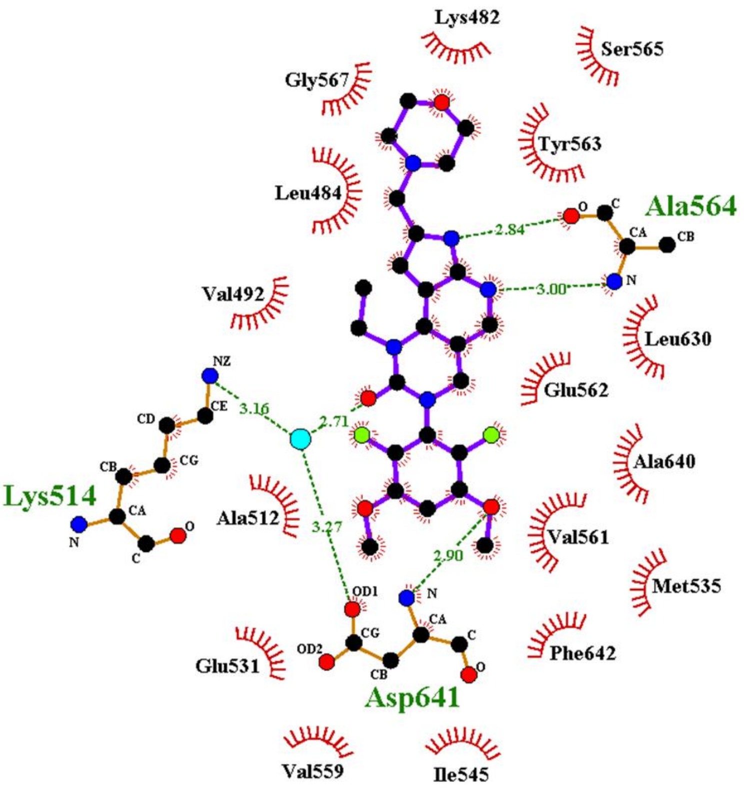

Supplementary Figure 1. Ligand interaction diagram (LID) demonstrated the interaction of FGFR1/Pemigatinib. Hydrogen bonds (distance ≤ 3.35 Å) are designated with a green dashed line, and van der Waals interactions (distance ≤ 3.9 Å) are shown by a red half "sun" arrangement. The water is showed as blue sphere. Graphics were drawn by LigPlot+.

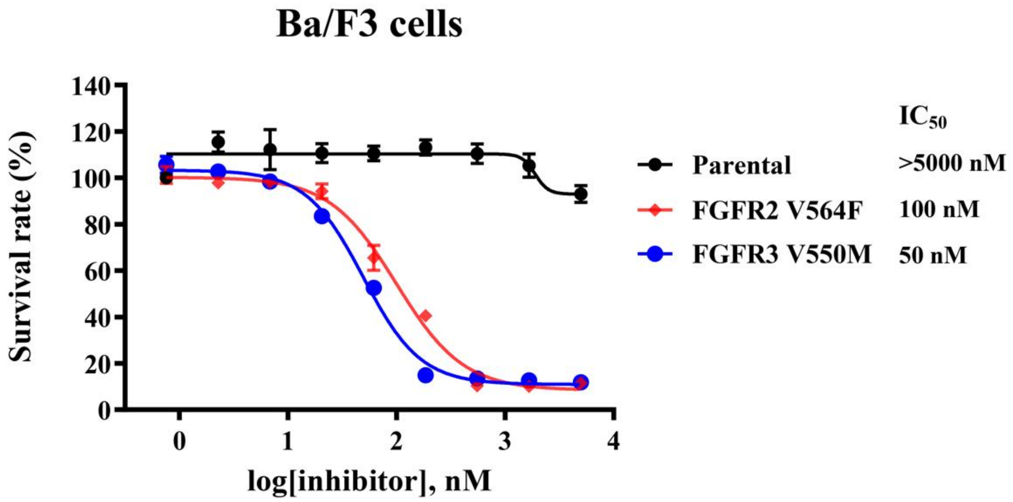

Supplementary Figure 2. Inhibitory effects of pemigatinib toward FGFR2 V564F and FGFR3 V555M gatekeeper mutants using a cellular proliferation assay. All error bars represent the standard deviation for at least three independent measurements.

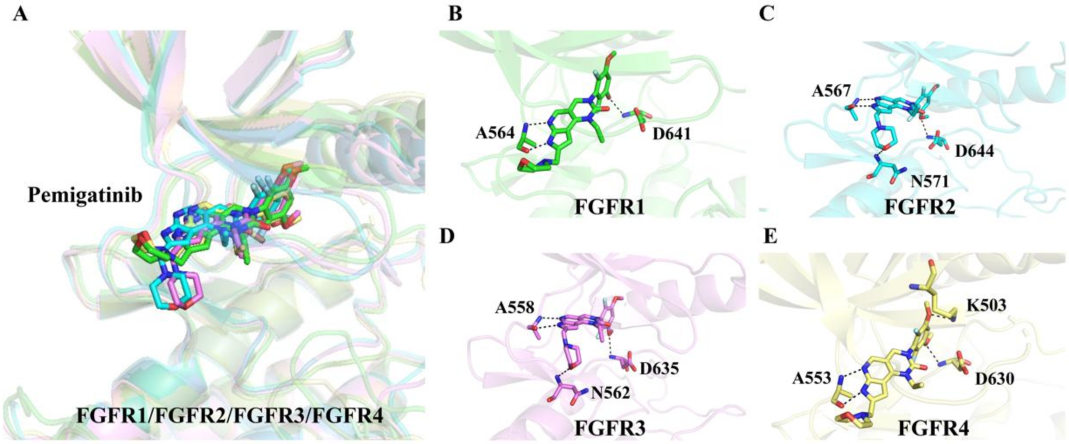

Supplementary Figure 3. Structural comparison of pemigatinib in complex with FGFR1-4. (A) Superposition of these structures. (B) Hydrogen-bond interactions between pemigatinib and FGFR1 (green). (C-E) Predicted hydrogen-bond interactions between pemigatinib and FGFR2 (cyan)/FGFR3 (magenta)/FGFR4 (yellow). These hydrogen-bond interaction patterns were analyzed by LigPlot+ and depicted by PyMOL. FGFR2/pemigatinib, FGFR3/pemigatinib and FGFR4/pemigatinib are predicted by docking pemigatinib into FGFR2 (PDB ID: 6LVL), FGFR3 (PDB ID:7DHL) and FGFR4 (PDB ID: 7F3M).

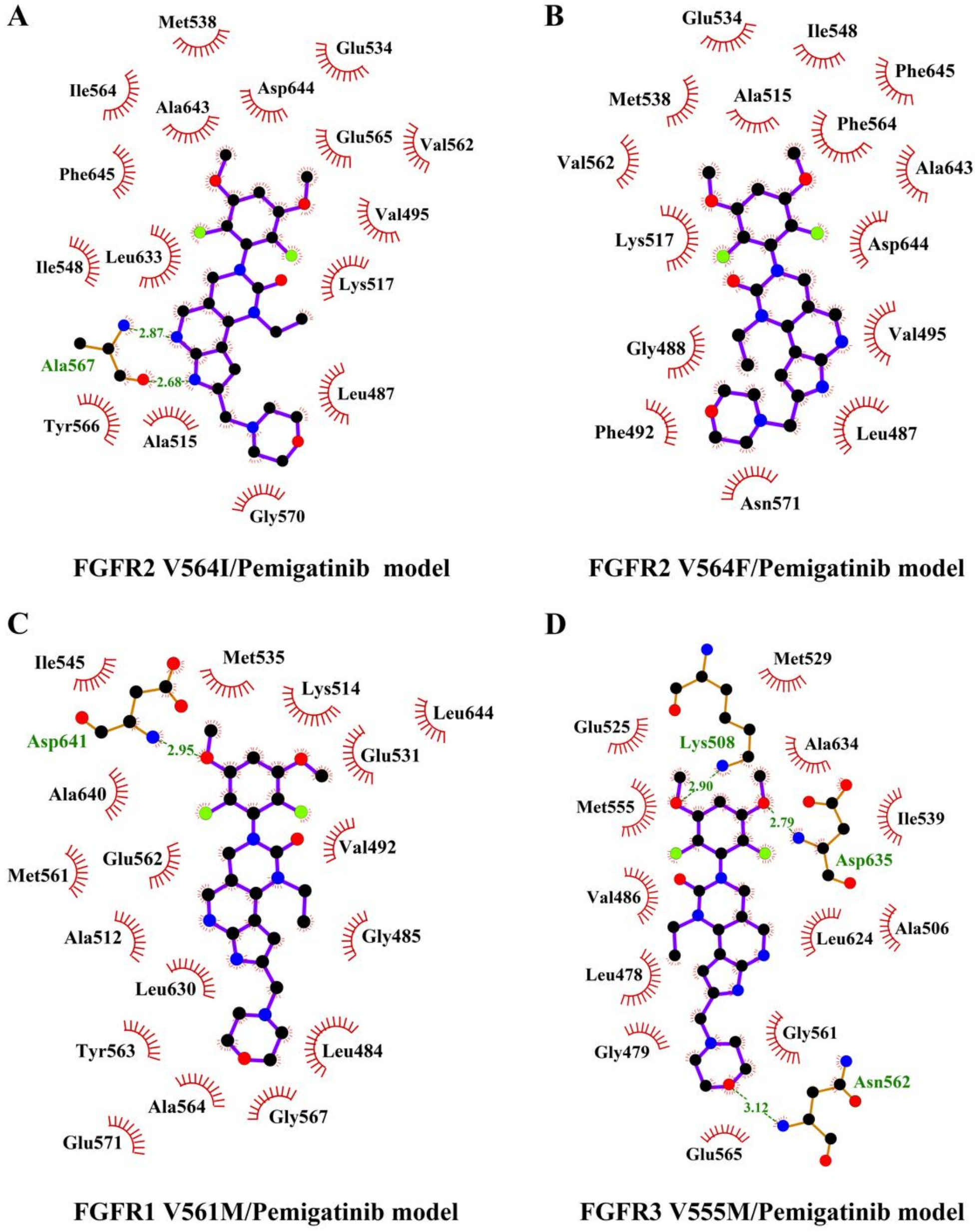

Supplementary Figure 4. Ligand interaction diagram (LID) demonstrated the interaction of pemigatinib with FGFR gatekeeper mutants. (A) Docking of the pemigatinib/FGFR2 V564I complex, the interaction pattern is similar to that of pemigatinib/FGFR2. (B) Docking of pemigatinib/FGFR2 V564F. (C) Docking of pemigatinib/FGFR1 V561M. (D) Docking of pemigatinib/FGFR3 V555M. Hydrogen bonds are designated with a green dashed line, and van der Waals interactions are shown by a red half "sun" arrangement. Graphics were drawn by LigPlot+.

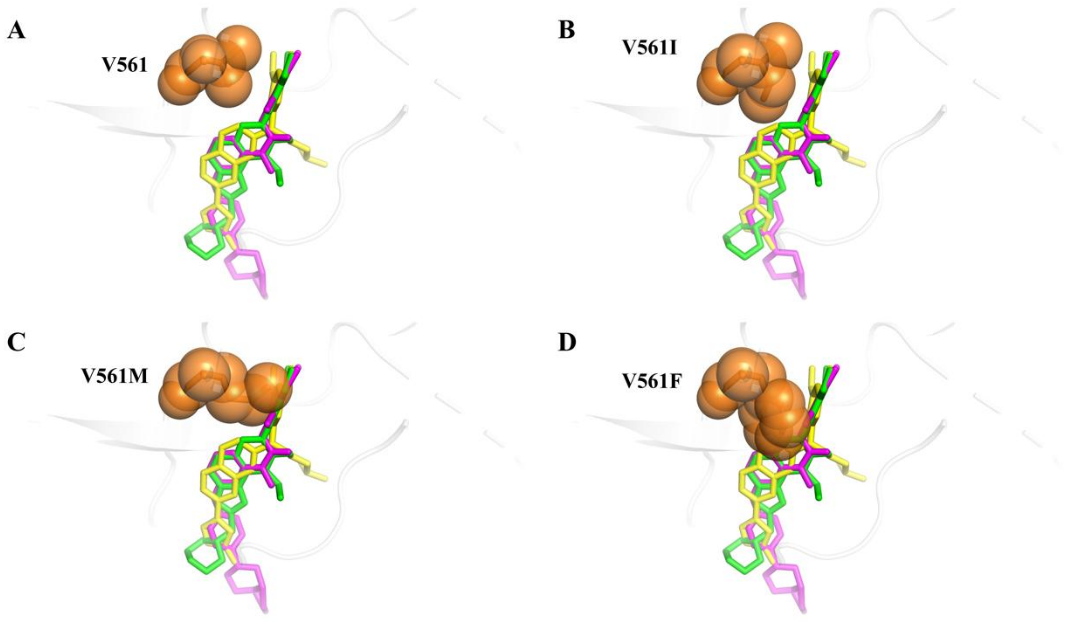

Supplementary Figure 5. Structural models of wild-type FGFR1 and FGFR1 V561I/M/F with pemigatinib/erdafitinib/infigratinib. (Green: pemigatinib; magenta: infigratinib; yellow: erdafitinib). (A) Superimposition of pemigatinib, erdafitinib and infigratinib in complex with wild-type FGFR1. (B) Superimposition of pemigatinib, erdafitinib and infigratinib in complex with FGFR1 V561I. (C-D) Overlay of pemigatinib, erdafitinib and infigratinib in complex with FGFR1 V561M/F. Amino acid residues corresponding to mutations show different responses to these three compounds. The structural models of FGFR1 V561I/M/F in complex with pemigatinib/erdafitinib/infigratinib are generated by substitution of gatekeeper residues on the basis of FGFR1/pemigatinib (PDB: 7WCL), FGFR1/erdafitinib (PDB: 5EW8) and FGFR1/infigratinib (PDB: 3TT0) structures.

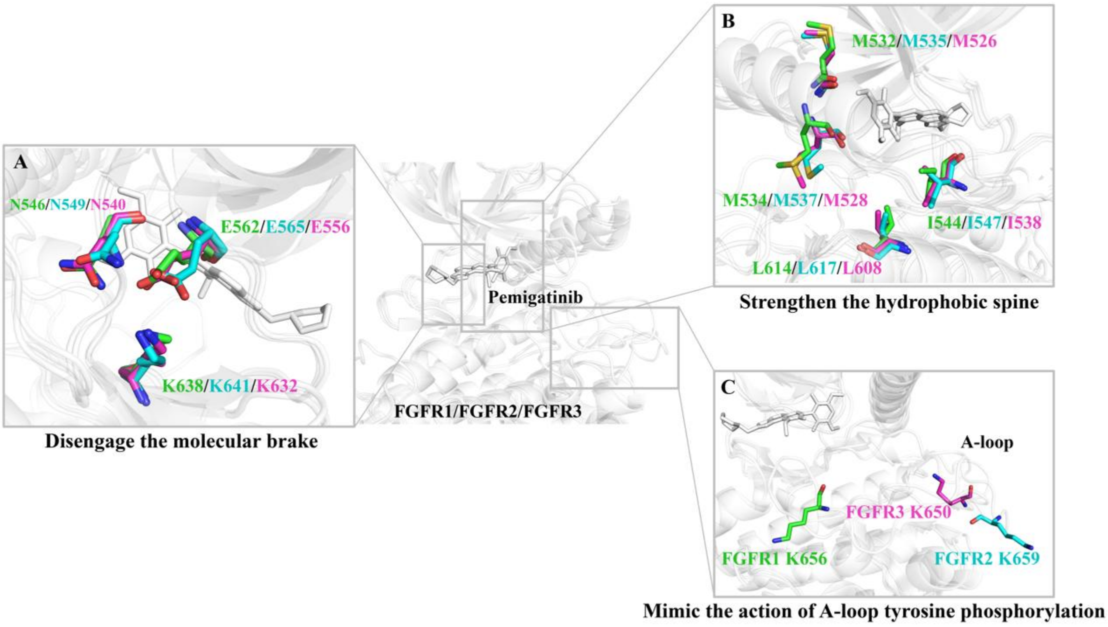

Supplementary Figure 6. The molecular mechanisms by which FGFR1-3 mutations may confer resistance to pemigatinib. (A) Mutations at FGFR1 N546/E562/K638, FGFR2 N549/E565/K641 or FGFR3 N540/E566/K632 may confer pemigatinib resistance by activating the kinase through disengagement of the molecular brake. (B) Some mutations may confer pemigatinib resistance by activating the kinase through strengthening the hydrophobic spine. (C) Mutations at FGFR1 K656, FGFR2 K659 or FGFR3 K650 may confer pemigatinib resistance by activating the kinase through mimicking the action of A-loop tyrosine phosphorylation. These residues are all conserved in FGFR1-3. FGFR1 residues are colored green; FGFR2 residues are colored cyan; FGFR3 residues are colored magenta.
