# Supplementary material for: Characterization of the cholangiocarcinoma drug pemigatinib against FGFR gatekeeper mutants
Source: Commun Chem. 2022 Aug 22;5:100. doi: 10.1038/s42004-022-00718-z (PMC9814635; doi:10.1038/s42004-022-00718-z)
Supplement: Supplementary file 3 — Description of Additional Supplementary Files [file 42004_2022_718_MOESM3_ESM.docx]

Description of Additional Supplementary Files

**File name: Supplementary Data 1**

**Description: Validation report of the PDB**
